# Microvalve Bioprinting of MSC-Chondrocyte Co-Cultures

**DOI:** 10.3390/cells10123329

**Published:** 2021-11-27

**Authors:** Joseph Dudman, Ana Marina Ferreira, Piergiorgio Gentile, Xiao Wang, Kenneth Dalgarno

**Affiliations:** 1 School of Engineering, Newcastle University, Newcastle upon Tyne NE3 1PS, UK; joedudman@outlook.com (J.D.); ana.ferreira-duarte@ncl.ac.uk (A.M.F.); piergiorgio.gentile@ncl.ac.uk (P.G.); 2 Translational and Clinical Research Institute, Medical School, Newcastle University, Newcastle upon Tyne NE2 4HH, UK; x.n.wang@ncl.ac.uk

**Keywords:** biofabrication, bioprinting, drop-on-demand, microvalve, micro-tissue, 3D cell culture, autologous chondrocyte implantation

## Abstract

Recent improvements within the fields of high-throughput screening and 3D tissue culture have provided the possibility of developing in vitro micro-tissue models that can be used to study diseases and screen potential new therapies. This paper reports a proof-of-concept study on the use of microvalve-based bioprinting to create laminar MSC-chondrocyte co-cultures to investigate whether the use of MSCs in ACI procedures would stimulate enhanced ECM production by chondrocytes. Microvalve-based bioprinting uses small-scale solenoid valves (microvalves) to deposit cells suspended in media in a consistent and repeatable manner. In this case, MSCs and chondrocytes have been sequentially printed into an insert-based transwell system in order to create a laminar co-culture, with variations in the ratios of the cell types used to investigate the potential for MSCs to stimulate ECM production. Histological and indirect immunofluorescence staining revealed the formation of dense tissue structures within the chondrocyte and MSC-chondrocyte cell co-cultures, alongside the establishment of a proliferative region at the base of the tissue. No stimulatory or inhibitory effect in terms of ECM production was observed through the introduction of MSCs, although the potential for an immunomodulatory benefit remains. This study, therefore, provides a novel method to enable the scalable production of therapeutically relevant micro-tissue models that can be used for in vitro research to optimise ACI procedures.

## 1. Introduction

Osteoarthritis is a chronic disease of the articular joint that results in the progressive degradation of cartilage and bone tissue as a consequence of a combination of pathophysiological mechanisms. Estimates of the impact of osteoarthritis on the global population calculated that in 2010 the disease was responsible for approximately 17 million years lived with disability, characterised by an increase of 64% since 1990 [1]. Current treatment strategies range from the application of holistic and self-management approaches to pharmacological therapies, with surgical techniques such as mosaicplasty or total knee replacement considered as the disease progresses [2].

A range of novel therapies is also being developed to prevent the onset of osteoarthritis including anti-TNFα therapy and new approaches to the established autologous chondrocyte implantation (ACI) technique [3,4,5]. ACI is a surgical technique that treats cartilage injuries to prevent the development of OA. ACI is typically performed by removing a small sample of cartilage from a reduced weight-bearing area of the affected joint. Chondrocytes are then isolated from the sample and expanded via in vitro monolayer culture. Once a sufficient number of cells have been cultured, the chondrocytes are implanted at the defect site using a periosteal flap or commercially available collagen membrane, with the implanted chondrocytes stimulating repair of the damaged tissue. More recent generations of ACI surgery include matrix-induced autologous chondrocyte implantation whereby cells are loaded onto a membrane prior to implantation, alongside techniques that involve the implantation of tissue-engineered cartilage cultured in vitro using autologous cells [6,7,8]. A minimum cell density of approximately 1 million cells per cm^2^ is commonly used during ACI procedures [9]. Recent research has examined the impact of combined autologous chondrocyte and bone marrow-derived mesenchymal stromal cell (MSC) implantation at the defect site, with the hypothesis being that the involvement of the MSCs will improve the repair, through their chondrogenic potential, and/or through the secretion of soluble factors which suppress immune reactions and stimulate endogenous stromal cells [10].

Increasing attention is being directed towards the development of high-throughput screening platforms for the assessment of new therapeutics at the preclinical level due to the high costs and low success rates associated with pharmaceutical research and development [11,12]. Of these platforms, 3D cell culture techniques are of significant interest due to their superior ability to recapitulate the internal conditions within the body when compared with commonly employed monolayer culture techniques [13,14,15,16]. Such models have applications for both pathophysiological disease research and therapeutic screening by providing a methodology to increase the throughput of early-stage research whilst simultaneously reducing the costs associated with therapeutic development. Despite this, 3D cell culture techniques increase the time, cost, and complexity of setting up and analysing models, thus limiting adoption within commercial environments that require high-throughput screening [17,18,19,20,21].

The field of biofabrication encompasses a range of novel technologies to selectively deposit cells and biological materials in order to generate cell-laden structures. These technologies have the potential to scale up the manufacture of in vitro cellular models by automating the deposition process. The most common cell printing techniques include material extrusion, inkjet, microvalve, and laser-based deposition processes. Each technique possesses unique advantages and disadvantages that are inherent to the actuation technology used.

Laser-based systems are capable of printing cellular material at high resolutions, but development is limited due to the high cost and complexity associated with the incorporated optical equipment [22,23,24]. Alternatively, material extrusion processes are able to deposit relatively high viscosity materials (normally gels) across a wide range of cell concentrations and rheological properties, providing a versatile method to dispense biological material [25]. As a result, a large variety of commercial bioprinting platforms have been recently developed for cell printing applications. However, the actuation process has been shown to induce higher pressures on the ink suspension than other techniques, with additional shear forces occurring during the final stages of printing [26]. In addition, the resolution currently attainable with such methods is significantly lower than that of other bioprinting technologies [27].

Inkjet and microvalve printing techniques provide a scalable, high-resolution method to deposit cells in low viscosity solutions. Inkjet printing processes can deposit material at the picolitre-scale, enabling the controlled deposition of single cells in media [28], but limitations of this technique include the limited printable cell concentration range that can be deposited, as well as reliability issues associated with nozzle clogging [29,30].

In contrast, microvalve printing has been successfully demonstrated at the nanolitre-scale for reliable cell printing of high cell concentration solutions [31,32]. Furthermore, previous research has explored the potential of using microvalve printing techniques to construct a range of micro-tissues [33,34,35,36].

Our aim in this work was to evaluate the potential for the use of microvalve-based bioprinting combined with transwell plates to provide a novel high throughput method for the production of chondrocyte-MSC co-cultures. Previous research into bioprinting for cartilage repair has predominantly focused on the production of chondral or osteochondral implants [37], whereas here we specifically focus on an approach with potential for high throughput evaluation of cell-based therapies exploiting the transwell approach.

Microvalve printing techniques employ a solenoid valve to control the rate of material flow from a pressurised reservoir. These devices can be operated in either a continuous jetting or drop-on-demand (DoD) configuration. Under a continuous jetting configuration, the volume of material ejected is controlled by adjusting the length of time that the valve is held open at a specified coordinate. Under a DoD configuration, the valve is sequentially opened and closed to deliver a finite number of droplets of material, with total deposition volume controlled by adjusting the number of actuation cycles at each position. Both approaches enable the deposition of material at the nanolitre-scale, with DoD printing enabling greater control of material ejection characteristics and in situ reactions at the expense of an increased valve duty cycle [31,38]. Trade-offs of the valve-printing process include the reduced maximum printing resolution and lack of modularity in controlling droplet ejection conditions when compared to inkjet printing [38,39,40].

Here, we present a bioprinted co-culture that can be used to investigate the effects of the relative cell number or additional biomolecular components in an MSC-chondrocyte co-culture within an insert-based culture format. This novel methodology incorporates a 96-well plate footprint to maintain compatibility with high-throughput culture techniques, thus providing a platform for future research into cell therapies for articular cartilage defects.

## 2. Materials and Methods

### 2.1. Cell Culture

Y201 hTERT immortalised human MSCs were cultured in high glucose (4.5 g/L) Dulbecco’s Modified Eagle Medium (DMEM, Thermo Fisher Scientific, Waltham, MA, USA) supplemented with 10% foetal bovine serum (Thermo Fisher Scientific), 100 U/mL Penicillin/100 µg/mL Streptomycin (Sigma-Aldrich, Gillingham, UK) and 2 mM L-Glutamine (Sigma-Aldrich). TC28a2 human chondrocytes (Merck, Gillingham, UK) were cultured in high glucose (4.5 g/L) DMEM/Ham’s F12 mix 1:1 *v*/*v* (Thermo Fisher Scientific) supplemented with 10% foetal bovine serum (Thermo Fisher Scientific), 100 U/mL Penicillin/100 µg/mL Streptomycin (Sigma-Aldrich) and 2 mM L-Glutamine (Sigma-Aldrich). Both cell lines were incubated at 37 °C, 5% CO_2_ prior to cell printing experiments.

### 2.2. Model Construction

**Figure 1:**
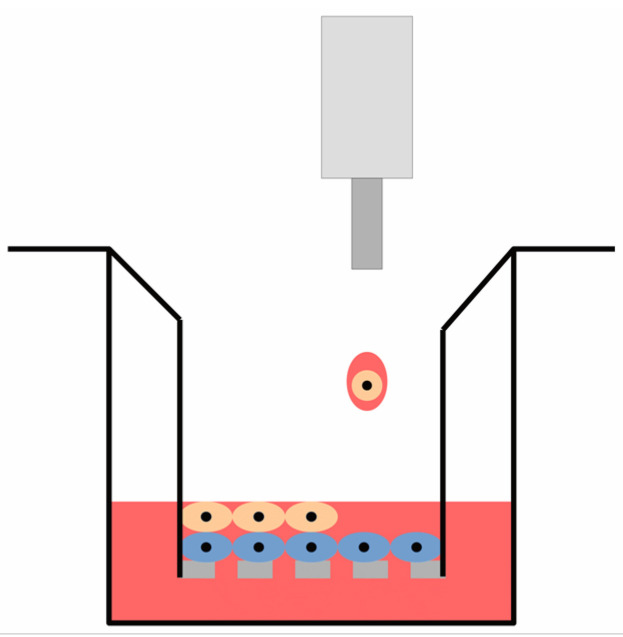
Illustration of the co-culture cell printing configuration using the Transwell^®^ insert culture platform. Chondrocytes were printed directly onto the surface of the permeable insert membrane followed by MSCs.

A Transwell^®^ 96-well plate permeable support system (Corning, Corning, NY, USA) was selected for the co-culture and consisted of polycarbonate well inserts with a 0.4 μm pore size. The inserts were pre-soaked in cell culture media prior to cell printing to promote cell attachment. Single cell-type cultures were printed by seeding cells directly onto the base of each insert. Co-culture samples were generated by seeding an equivalent number of chondrocytes immediately followed by MSCs onto the base of each insert, as shown in Figure 1. A total of 143,000 cells were seeded onto the insert within an 80 µL volume of DMEM/F12 cell culture media to achieve a cell seeding density equivalent to 1 million cells per cm^2^. A 235 µL volume of DMEM/F12 cell culture media was also added to each receiver plate well and media was exchanged daily in both compartments of the permeable support system.

### 2.3. Printing Configuration

A JetLab^®^ 4 XL (MicroFab, Plano, TX, USA) printing workstation combined with the JetDrive^®^ printer drive electronics unit was used to print the co-cultures. The system was controlled using coordinate-based jetting commands to deliver variable volumes of material within each insert well according to the programmed number of actuation cycle requests.

The device was cleaned by flushing 1% *v*/*v* Micro-90^®^ cleaning solution (Cole-Parmer, St. Neots, UK) followed by 70% *v*/*v* ethanol (Fisher Scientific) through the reservoir, tubing and jetting device immediately prior to and following printing to remove any potential sources of contamination.

Cells suspended in a culture medium were used as the bio-ink in each of the bioprinting experiments. Briefly, the cell culture medium was warmed to 37 °C and filtered using a 0.22 µm filter (Merck) prior to use. Then, the cells were suspended into filtered cell culture media and centrifuged at 200× *g* for 5 min. The supernatants were discarded, and the pellets were resuspended within the culture media formulation and transferred to the reservoir for printing.

Bio-inks were deposited using VHS Series 24 V solenoid valves connected via a 062 MINSTAC^®^ fitting to a 0.25 mm jewelled orifice dispensing nozzle (The Lee Company, USA). An adjustable spike-and-hold driver (The Lee Company) was used to convert the JetDrive^®^ waveform output into a valve-compatible actuation signal to modulate device actuation under a DoD configuration. A jetting frequency of 500 Hz was configured alongside a valve open time of 200 µs to deliver cellular material. An external power supply (ISO-Tech, UK) was connected to the spike-and-hold driver to provide a continuous 24 V supply to the spike driver terminal and a 5 V supply via a separate channel to the hold driver terminal. Backpressure of 200 mmHg was applied to the reservoir during printing using a MicroFab CT-PT4 standalone pneumatics controller box.

**Figure 2:**
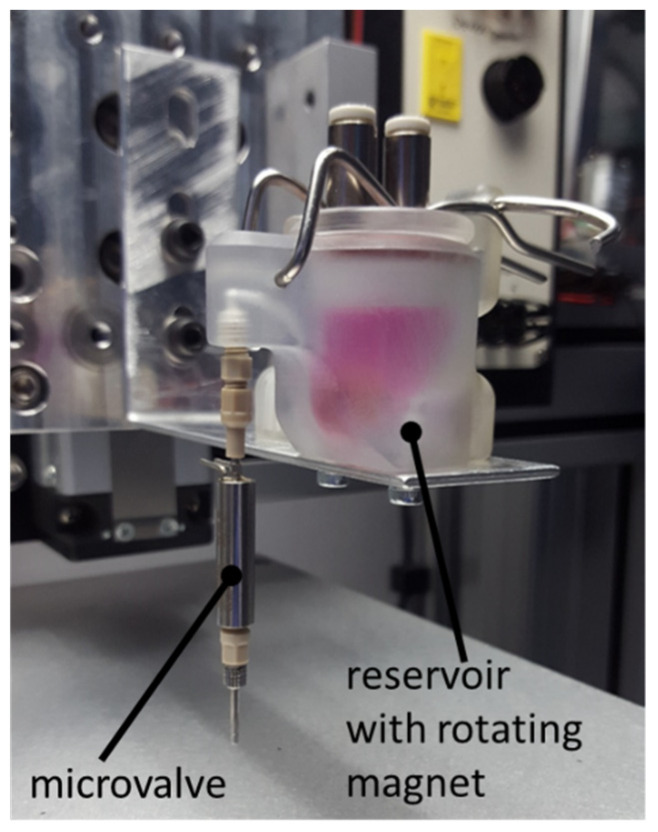
Printer configuration showing the microvalve actuator attached to the agitated reservoir. Following ink loading, a small cylindrical magnet is placed within the ink suspension and the lid clamped shut. The magnet is rotated using a motor mounted externally to the ink compartment and pressure maintained via the top-mounted air inlet ports.

A custom reservoir device was used for all cell printing experiments to maintain cellular dispersion within the ink suspension. The sealed device featured an electrical motor coupled to a magnet external to the reservoir that induced rotation of a gold-plated cylindrical neodymium magnet located on the inside of the reservoir. The microvalve was mounted directly to the reservoir to minimise the volume of ink held in connecting tubing, as shown in Figure 2.

### 2.4. Characterisation of Printing Performance

The impact of printing parameters on cell deposition performance was determined by counting the number of cells contained within single droplets printed onto a glass slide. Cells were printed at an ink concentration of 10^6^ cells per mL of cell culture media and immediately counted using a DMLB microscope (Leica Biosystems, Wetzlar, Germany).

Microvalve dispensing performance was characterised gravimetrically by depositing DMEM cell culture media onto a glass substrate. Liquid mass was measured immediately following printing using an Explorer^®^ analytical balance (Ohaus, Parsippany, NJ, USA).

The impact of ink cell concentration on printing performance was determined by printing material into the individual wells of a multi-well plate. A 10 μL aliquot of the cell suspension was then immediately transferred to a 0.1 mm depth Neubauer chamber haemocytometer (Hawksley, Lancing, UK) for cell counting.

### 2.5. Cell Viability Assay

A Live/Dead^®^ cell viability assay (Thermo Fisher Scientific) was performed to evaluate the impact of the printing processes on the cellular viability of the MSC and chondrocyte cell types. Cells were printed or manually pipetted onto glass coverslips at a concentration of 10^6^ cells per mL and assessed both immediately following printing and after a 24 h incubation period. Stock solutions were warmed to room temperature and diluted in PBS (Sigma-Aldrich) to produce a 2 μM calcein acetoxymethyl and 4 μM ethidium homodimer-1 working solution. Adherent cells were washed extensively with PBS prior to the application of a Live/Dead^®^ working solution. The samples were incubated for 30 min in a humidified atmosphere at 37 °C, 5% CO_2_ prior to imaging. Imaging was performed using a DMLB fluorescence microscope (Leica Biosystems) at 100× magnification, and images were captured using a SPOT Advanced CMOS camera (Spot Imaging Solutions, Sterling Heights, MI, USA) and corresponding microscopy software.

### 2.6. Metabolic Activity Assay

The impact of the printing process on the metabolic activity of each cell line was determined using a PrestoBlue^®^ assay. Cells were seeded into the wells of a 96-well plate at a concentration of 10^6^ cells per mL using microvalve printing or manual pipetting. Following incubation for each time point, the supernatant from each well was removed and replaced with 100 µL of PrestoBlue^®^ (Thermo Fisher Scientific) working solution previously prepared via the addition of 10% *v*/*v* PrestoBlue^®^ reagent to phenol-red free cell culture media. Plates were incubated for 4 h and fluorescence values were obtained using a FLUOstar^®^ Omega microplate reader (BMG Labtech, Ortenberg, Germany) at an excitation and emission filter of 544 nm and 620 nm, respectively.

### 2.7. Visualisation of Cell Morphology

Cell morphology was visualised in 2D using a combination of phalloidin–tetramethylrhodamine B isothiocyanate (Sigma-Aldrich) and 4′,6-diamidino-2-phenylindole (DAPI; Sigma-Aldrich) fluorescence stains to indicate cytoskeletal filamentous actin and cell nuclei, respectively. Cells were printed or manually pipetted onto glass coverslips at a concentration of 10^6^ cells per mL, which were then incubated for each time point and subsequently fixed in 4% paraformaldehyde solution (Thermo Fisher Scientific) for staining. Fixed cells were washed extensively in PBS prior to the addition of 1 μg/mL phalloidin working solution prepared in PBS. Cells were incubated for 20 min at room temperature prior to three washes in PBS. Coverslips were then mounted onto glass slides via the addition of Fluoroshield™ with DAPI mounting medium (Sigma-Aldrich) and visualised after a 10 min incubation period using an LSM800 point scanning confocal microscope (Carl Zeiss AG, Jena, Germany) at 20× magnification.

### 2.8. Cell Proliferation Rate

The proliferation rates of the MSC and chondrocyte cell lines were calculated following printing using a trypan blue exclusion test. Cells were either printed or manually pipetted at a concentration of 10^6^ cells per mL into the wells of a six-well plate containing cell culture media. At each respective time point, cells were trypsinised and a 10 μL aliquot combined with an equal volume of trypan blue staining solution (Sigma-Aldrich) prior to counting using a haemocytometer.

### 2.9. Membrane Sectioning

Samples were isolated at each time point by removal of the insert from the Transwell^®^ receiver plate. Membranes were separated from the insert using a scalpel and embedded in Tissue-Tek^®^ optimal cutting temperature compound (Sakura Finetek Europe, Alphen aan den Rijn, Netherlands). Samples were snap-frozen in liquid nitrogen until solid and were kept at −80 °C until cryosectioning. Samples were cryosectioned using a CM1900 cryostat (Leica Biosystems) at a thickness of 10 μm. Slides were kept at −80 °C until analysis, where they were dried, sections restricted with a PAP pen (Sigma-Aldrich), washed in PBS, and fixed for 30 min in 4% paraformaldehyde solution (Thermo Fisher Scientific).

### 2.10. Haematoxylin and Eosin Staining

Optimal cutting temperature compound embedded sections were immersed in PBS for approximately 1 min prior to staining. Samples were incubated in Mayer’s haematoxylin (Sigma-Aldrich) for 5 min prior to rinsing in running tap water for 2 min to blue haematoxylin-stained nuclei. Samples were dehydrated through incubation in ethanol solutions of increasing concentration prior to immersion in eosin solution (Sigma-Aldrich) for 30 s. Samples were further dehydrated and incubated in Histo-Clear^®^ II (National Diagnostics, USA) for 5 min before being mounted in DPX (Sigma-Aldrich). When dry, samples were imaged using an Axio Imager^®^ (Carl Zeiss AG) upright microscope at 20× magnification.

### 2.11. Indirect Immunofluorescence Staining

Fixed samples were permeabilised in 0.1% Triton X (Merck) and washed in PBS 3 times for 5-min intervals. Samples were then blocked with 2% bovine serum albumin (Merck) in PBS for 30 min at room temperature. Excess bovine serum albumin was removed and replaced with a primary antibody solution. Primary antibodies were diluted in PBS to achieve a final concentration of 1:200 for Collagen II (ab34712; Abcam, Cambridge, UK) and 1:50 for Aggrecan (ab3778, Abcam). Samples were incubated in the antibody solution for 2 h at room temperature before being washed 3 times for 5 min in PBS. A secondary antibody solution was then prepared using Alexa Fluor^®^ 488 or 594 (Thermo Fisher Scientific) at a concentration of 1:500 and added to the samples for further 1-h incubation at room temperature in the dark. Samples were washed three times in PBS and mounted in Fluoroshield^®^ with DAPI mounting medium (Sigma-Aldrich) containing DAPI nuclear counterstain. Imaging was performed using an LSM800 point scanning confocal microscope (Carl Zeiss AG) at 200× magnification.

### 2.12. Cell Tracker Staining

CellTracker^®^ Red and CellTracker^®^ Green dyes (Thermo Fisher Scientific) were used to label MSC and chondrocyte cell samples, respectively, prior to printing. Cell Tracker^®^ solutions were prepared to a final concentration of 25 µM in cell culture media. Pelleted cells were resuspended in the CellTracker^®^ solutions and incubated for 45 min at 37 °C in the dark. Following centrifugation, the supernatant from each sample was removed and cells resuspended in cell culture media for printing. At the desired time points, the Transwell^®^ membranes were removed from the insert mounts, fixed for 30 min in 4% paraformaldehyde solution (Thermo Fisher Scientific), and washed three times in PBS (Sigma-Aldrich). Whole samples were mounted onto glass slides using DPX (Sigma-Aldrich) prior to imaging. Labelled samples were imaged using an LSM800 point scanning confocal microscope (Carl Zeiss AG) at 20× magnification.

### 2.13. Model Cell Proliferation Assay

Cell proliferation within the co-cultures was assessed using the 5-ethynyl-2′-deoxyuridine (EdU)-Click 594 cell proliferation assay (Baseclick, Neuried, Germany). Samples were seeded and cultured in media containing 10 µM EdU for 48 h prior to processing at each time point. Day 1 samples were incubated in EdU for 24 h. Transwell^®^ inserts were then snap-frozen and sectioned as previously described. Sections were stained with the EDU-Click kit according to the manufacturer’s instructions and mounted in Fluoroshield^®^ mounting medium (Sigma-Aldrich) containing DAPI nuclear counterstain. Imaging was performed using an LSM800 point scanning confocal microscope (Carl Zeiss AG) at 20× magnification.

### 2.14. Statistical Analysis

Data presented show mean values ± standard deviation. Data were analysed using Prism^®^ 8 statistical analysis software (GraphPad Software, USA) using two-way analysis of variance in combination with Tukey or Šídák multiple comparison tests unless stated otherwise. Levels of statistical significance were defined using *p* ≤ 0.05 (*), *p* ≤ 0.01 (**) and *p* ≤ 0.001 (***). Microvalve dispensing performance was assessed via linear regression analysis.

Cell proliferation assays were assessed via nonlinear regression analysis using an exponential (Malthusian) population growth model defined by:

(1) $$Y=Y_{0}e^{\mathrm{kx}}$$

where Y_0_ is the starting population, k is the rate constant, and x is the population doubling time.

An extra sum-of-squares F test was performed to compare the rate constant value of each proliferation assay sample group.

## 3. Results

### 3.1. Microvalve Printing of MSCs and Chondrocytes

**Figure 3:**
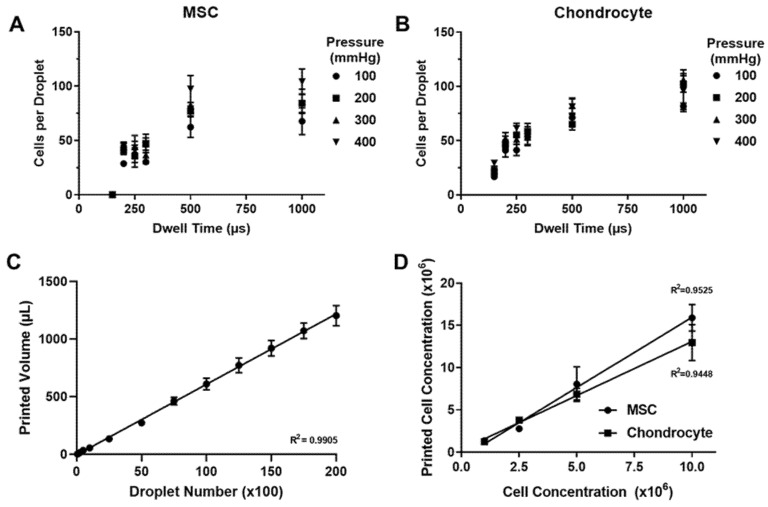
Dispensing performance of the microvalve printing platform under a drop-on-demand configuration. (**A**) Influence of waveform dwell time and backpressure applied to valve on cell density per droplet when printing MSCs at a concentration of 10^6^ cells per mL. (**B**) Influence of waveform dwell time and backpressure applied to valve on cell density per droplet when printing chondrocytes at a concentration of 10^6^ cells per mL. (**C**) Dispense volume per droplet number. The gradient of the linear regression trend line corresponds to a droplet volume of 61 nL. (**D**) Impact of ink cell concentration on cell printing performance. Data represent mean values ± SD. N = 3–6.

Printing performance was characterised across a range of actuation signal dwell times and backpressures to determine an optimal cell printing configuration, as shown in Figure 3. An increase in cell number per droplet was observed in response to increases in the actuation signal dwell time. This effect was diminished beyond a dwell time of 500 μs, with a minimum of 200 μs and 150 μs required to deposit cellular material for the MSC and chondrocyte cell lines, respectively. Pressure adjustments were shown to have no significant impact on cell printing performance when compared via linear regression analysis. A dwell time of 200 μs and a pressure of 200 mmHg was selected for printing experiments to maximise the resolution of the microvalve printing process whilst maintaining printing reliability.

The jetting performance of the microvalve was benchmarked across a range of deposition volumes, as displayed in Figure 3. A high degree of linearity was observed between the actuation cycle number and total ejection volume, with linear regression analysis indicating a droplet volume of approximately 60 nL. The percentage coefficient of variation was calculated to be below 12% across the deposition range assessed.

The effect of ink cell concentration on printing performance was also assessed in Figure 3. A high degree of linearity was observed between the concentration of the ink in the printer reservoir and the concentration of printed material. Printing was achieved up to a cell concentration of 10^7^ cells per ml for both cell lines with linear regression analysis indicating a significant difference in printing performance between the MSC and chondrocyte cell lines.

**Figure 4:**
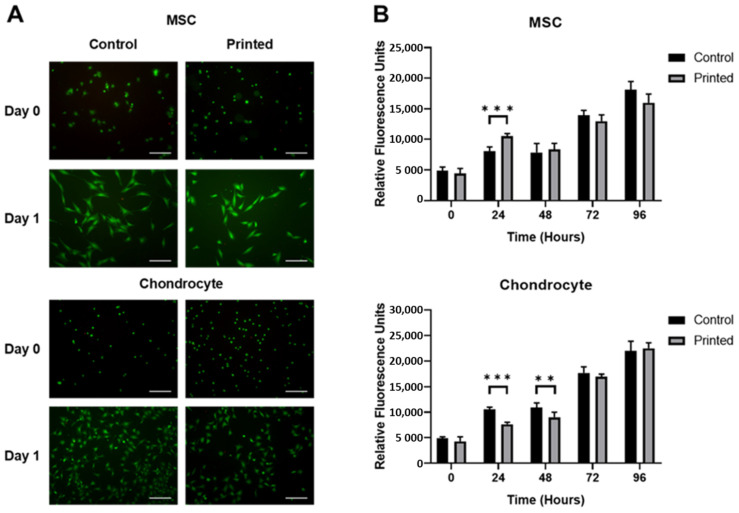
Effect of printing process on the viability and function of the MSC and chondrocyte cell lines printed at 10^6^ cells per mL. (**A**) Cell viability assay showing live (green) and dead (red) cells. Scale bar = 200 μm. (**B**) Metabolic activity assay. Data represent mean values ± SD. N = 6. Significance as outlined in Section 2.14.

A Live/Dead^®^ cell viability assay was used to assess the impact of the printing process on the cell viability of MSCs and chondrocytes in Figure 4. Cell viability assay data revealed no discernible differences in the ratio of live to dead cells when comparing manually pipetted and microvalve printed samples, both immediately following and 24 h after printing, for either cell line. At 24 h, both cell lines were shown to adhere to the substrate irrespective of the seeding method.

Figure 4 demonstrates that the impact of the printing process on the metabolic activity of each cell line was small. A small significant difference in metabolic activity was observed when comparing printed and manually pipetted MSCs at the 24 h time point, with a small significant difference between printed and manually pipetted chondrocytes also observed at both the 24 and 48 h time points. No significant differences in metabolic activity were observed as a result of the printing process either immediately following printing or throughout the remainder of the assessment period.

**Figure 5:**
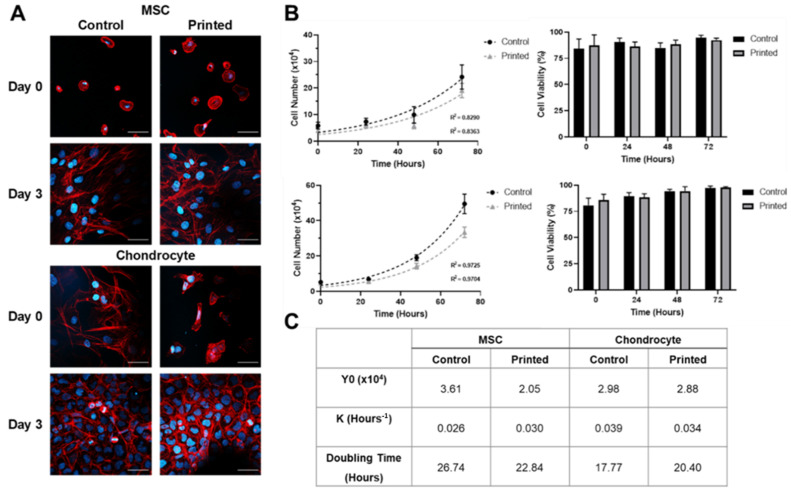
Comparison between the morphology and proliferative activity of printed and manually pipetted cells when deposited at concentrations of 10^6^ cells per mL. (**A**) Morphology of MSC and chondrocyte cell lines showing cell nuclei visualised using DAPI (blue) and filamentous actin using phalloidin (red) staining. Scale bar = 50 μm. (**B**) Proliferation rate of MSC and chondrocyte cell lines assessed via cell counting using a trypan blue dye exclusion test. (**C**) Non-linear regression analysis using an exponential growth model of cell proliferation. Data represent mean values ± SD. N = 6.

The impact of the printing process on cellular morphology and proliferation rate was assessed for both cell lines, as shown in Figure 5. No discernible differences in cell size, morphology, or adherence were observed across either cell line when comparing printed and manually pipetted samples. Cellular proliferation data were also collected, revealing no significant differences between the doubling time of printed versus manually pipetted samples.

### 3.2. MSC and Chondrocyte Co-Cultures

**Figure 6:**
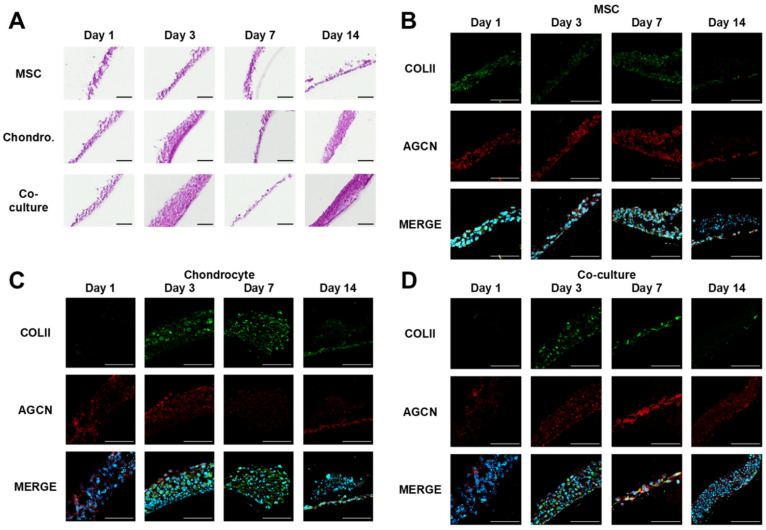
Histological characterisation of tissue model. Sections were oriented to display the Transwell^®^ membrane at the base of each image. (**A**) Haematoxylin and eosin staining of printed cultures generated from MSCs, chondrocytes and cell co-cultures over a 14-day culture period. (**B**–**D**) Immunostaining data comparing the collagen II (COLII, green) and aggrecan (AGCN, red) content of MSC, chondrocyte and MSC-chondrocyte printed cultures. Cell nuclei were visualised using DAPI (blue) staining. Scale bar = 100 μm.

The influence of the relative cell number on tissue formation and architecture was assessed via haematoxylin and eosin staining, as shown in Figure 6. Micro-tissues were generated within the first 24 h of culture when seeded onto the Transwell^®^ membrane inserts. Indiscriminate separation from the membrane occurred as a result of the sectioning process. Cell density was reduced towards the surface of each sample with the exception of the co-culture model analysed at day 14, which displayed two distinct layered regions indicative of cellular organisation.

Qualitative assessment of sectioned models in Figure 6 revealed an increase in collagen II protein expression within the chondrocyte and cell co-culture samples when compared with MSC-only tissue, particularly at days 3 and 7. The Y201 cell line shows the early expression of collagen II, which is normal for this line, but no increase over time. A low degree of aggrecan protein expression was observed across all time points, irrespective of cellular composition. In addition, variations in nuclear size were also observed within the chondrocyte and cell co-cultures following tissue formation, as evidenced within both haematoxylin and eosin and indirect immunofluorescence-stained samples.

**Figure 7:**
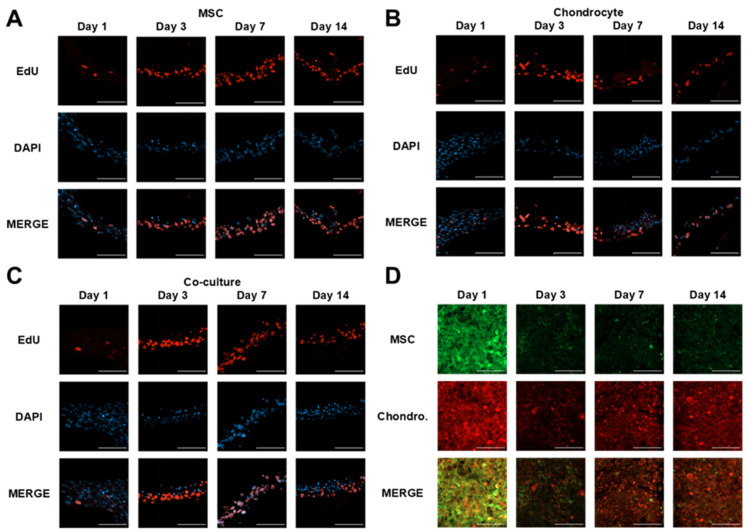
Cellular proliferation and localisation within tissue models. (**A**–**C**) Immunofluorescent detection of proliferating cells via EdU (5-Ethynyl-2′-deoxyuridine) incorporation within MSC, chondrocyte and MSC-chondrocyte co-cultures. Cell nuclei were visualised using DAPI (blue) staining. Sections were oriented to display the Transwell^®^ membrane at the base of each image. (**D**) Localisation of MSC (green) and chondrocyte (red) cells on the surface of the co-culture over a 14-day culture period. Cells were labelled using CellTracker^®^ fluorescent dyes prior to printing. Scale bar = 100 μm.

Assessment of the proliferation rate within each model revealed no discernible differences in the proportion of proliferating cells in response to variations in cellular composition, as indicated in Figure 7. From Day 3 onwards, proliferation was most prominent at the base of the tissue at the interface with the Transwell^®^ insert membrane.

Analysis of cellular composition within the co-culture samples was also performed in order to compare the ratio of MSCs to chondrocytes over the 14-day culture period, as shown in Figure 7. A reduction in CellTracker^®^ fluorescence signal intensity was observed for each cell line within the first 3 days of culture as a consequence of fluorophore degradation and externalisation. An elevation in chondrocyte signal intensity was observed at the upper surface of the co-culture model throughout the remaining time points.

## 4. Discussion

Micro-tissue models are of considerable interest for drug discovery and tissue engineering research due to their ability to recapitulate the tissue architecture, diffusion gradients, and cell-cell or cell-extracellular matrix contact of living tissue [41,42,43]. Previous studies have explored the application of bioprinting technologies such as microvalve printing for the construction of micro-tissue models. Within this research, we successfully demonstrated that microvalve bioprinting can be used to generate micro-tissues of MSC and chondrocyte cell types, providing a potential platform to study cell therapies for cartilage defect repair.

Initial investigations into the performance of the microvalve printing technique highlighted the impact of actuation signal dwell time on cell number per droplet, as shown in Figure 3. A minimum dwell time was established in order to successfully print each cell type through the ejection of material from the microvalve nozzle. Reservoir pressure was shown to have no significant impact on printing performance, contrasting with the findings of other research in this field [44]. We hypothesise that this was due to the low-pressure range studied, which we employed to minimise any potential pressure-induced damage to suspended cells. An actuation signal dwell time of 200 μs and pressure of 200 mmHg was selected for printing experiments to maintain printing reliability whilst maximising potential print resolution.

Microvalve printing processes typically provide less control over droplet ejection properties in comparison to inkjet printing due to the more limited adjustments that can be made to the actuation signal waveform [44,45,46]. In addition, this technology is often operated in a continuous jetting mode, whereby the valve is held open until a target volume was ejected in the form of a single stream of fluid. The data presented in Figure 3 provide an important validation step for the DoD printing approach, demonstrating the use of actuation cycle numbers to precisely control total deposition volume. Under this configuration, a high degree of linearity was observed between actuation cycle number and ejected droplet volume, enabling the sequential deposition of droplets with an individual volume of approximately 60 nL onto the printing substrate. Importantly, these findings demonstrate that the microvalve printing process can be used to sequentially deposit material at a resolution and accuracy that is competitive with state-of-the-art continuous jetting approaches, as well as superior to contact printing techniques, whilst retaining the depositional control of droplet-based approaches [38,47].

The microvalve printing process was also shown to be capable of depositing cells up to an ink cell concentration of 10^7^ cells per mL for both cell lines. A higher cell concentration was observed in printed material versus the ink cell concentration loaded into the printer reservoir, as shown in Figure 3. In addition, greater variability in printed cell concentration was observed at higher concentrations. These effects could have been caused by many factors, with localised cell sedimentation considered the most likely. However, Figure 4 demonstrates that once the system had been calibrated consistent cell numbers were printed and that these were comparable to pipetted controls. Microvalve printing did not adversely affect the viability or metabolic activity of either the chondrocytes or the MSCs (Figure 4 and Figure 5), and no changes in cell behaviour were observed.

In combination, these results provide important characterisation data for printing MSC and chondrocyte cell types using the microvalve printing process. Overall, both cell types were highly compatible with microvalve printing, confirming its suitability for the production of MSC and chondrocyte co-cultures.

The printing process was successfully used to generate cultures of the MSC and chondrocyte cell lines in both monoculture and co-culture. Models were generated using cell lines to provide a platform that could be used to research the impact of MSCs on cartilage tissue formation and chondrocyte cell therapies [5,10,48,49]. The intention here was not to create a cartilage tissue model, but broadly to replicate the cell seeding densities that would be delivered to a defect site in ACI, to assess in a simplified co-culture the effect of MSCs on chondrocytes.

Haematoxylin and eosin staining (Figure 6) demonstrated the formation of micro-tissues on the upper surface of insert membranes. An increase in tissue thickness was observed over time amongst chondrocyte and co-culture samples, providing evidence of active cell proliferation within the micro-tissue.

The extracellular matrix of hyaline cartilage tissue is predominantly comprised of collagen II and aggrecan, a proteoglycan. In combination, collagen II and aggrecan provide an integral role in enabling cartilage tissue to resist tensile and compressive forces [50,51]. Indirect immunofluorescence staining (Figure 6) revealed an elevation in collagen II expression amongst chondrocyte and co-culture samples during culture, providing evidence of the formation of ECM components by cultured chondrocytes. In this regard, the presence of MSCs in the co-culture seems to have had neither a positive or negative effect, suggesting that if MSCs offered an immune or endogenous stimulation effect in vivo that this would not be at the cost of inhibiting ECM production by chondrocytes.

Variations in nuclei size were observed throughout the cross-section of the chondrocyte and cell co-culture micro-tissues. These changes are characteristic of nuclear blebbing, a process that leads to structural changes which result in chromatin condensation and fragmentation [52]. From day 3 to 14, cells undergoing nuclear blebbing transitioned from being evenly dispersed within the tissue to being localised on the upper surface region of the co-culture. These results indicate a potential organisational response within both cell populations when co-cultured, supporting previous studies observing a relationship between MSC and chondrocyte co-cultures when compared to monocultures of each cell type [53,54].

Proliferation assay data (Figure 7) indicated that cellular proliferation was maintained across all models throughout the full 14-day culture period, with the cellular composition having no discernible impact on proliferation activity. Proliferation was shown to be most prominent at the base of the tissue on the interface with the Transwell^®^ insert membrane, perhaps as a result of cell adherence on the membrane and indicative of a polarised tissue structure whereby cells transitioned from the proliferative region at the base of the tissue to the upper surface, undergoing maturation and subsequent controlled cell death. These results corroborate with the histological staining (Figure 6), highlighting a reduction in cell density towards the upper surface of the tissue across each model, alongside changes in cell nuclei size and shape throughout the tissue cross-section.

CellTracker^®^ data (Figure 7) revealed an elevation in fluorescence signal intensity from day 3-14, indicative of the localisation of the chondrocyte cell type towards the upper surface of the tissue. A reduction in signal intensity was observed within the first 3 days of culture as a result of fluorophore degradation and reduced retention within proliferating cells. These results are supportive of the observed changes in nuclei size (Figure 6), indicating an organisational response within the co-cultured tissue.

Future work with the model will focus on:

1. Further validation of the physiological relevance of the model, using sets of donor-matched primary cells cultured as per ACI processes, and including further cell types relevant to the joint niche.
2. Using higher-throughput proteomic analysis techniques, such as supernatant analysis, and positional transcriptomic techniques such as MALDI-imaging, in order to add further depth to the analysis and take advantage of the highly scalable printed culture format.
3. Improvements to the cell culture platform that could enable further increases in cell culture density per well, the introduction of cytokines or other biomolecules, as well as address throughput limitations relating to the manual exchange of media during culture. A multi-microvalve platform could be developed to enable the simultaneous printing of multiple models. This has the potential to greatly increase printing throughput beyond approximately one model per second depending on the number of microvalves incorporated. Through increasing printing throughout, this modification would also reduce the total time that cells are held in suspension when printing significant numbers of models.

Overall, our findings demonstrate that microvalve bioprinting can be used to reliably print MSC and chondrocyte cell types in order to generate insert-based co-cultures. The observed increases in collagen II expression, proliferative activity, and the indication of an organisational response within the co-cultures provide a promising scalable model to research the impact of procedural variations on cellular therapies such as ACI.

## 5. Conclusions

Within this study, a DoD microvalve bioprinting process was used to generate MSC and chondrocyte cell co-cultures that could be used as a platform to study cell therapies for cartilage repair. The microvalve printing process was shown to be capable of reliably dispensing material across a wide volume range through the sequential deposition of 60 nL volume droplets. Microvalve deposition was shown to be compatible with both MSC and chondrocyte cell lines across a range of bio-ink cell concentrations, enabling the printing of cell co-cultures onto an insert-based culture platform. Characterisation of the printed co-cultures revealed that MSCs do not inhibit chondrocytes in terms of the production of collagen II and aggrecan and showed the formation of ECM alongside a proliferative region of cells at the base of the tissue. These results indicate a promising and scalable model that could be used for in vitro research into cartilage repair treatments such as ACI. Future work will focus on developing high throughput characterisation techniques and more complex co-cultures to allow a wider range of variables to be studied.

## Data Availability

Data supporting this publication are openly available under an ‘Open Data Commons Open Database License’. Additional metadata are available at https://doi.org/10.25405/data.ncl.12973109.v1 (accessed on 26 November 2021).
